# The Warburg Effect Is Associated With Tumor Aggressiveness in Testicular Germ Cell Tumors

**DOI:** 10.3389/fendo.2019.00417

**Published:** 2019-06-28

**Authors:** Murilo Bonatelli, Eduardo C. A. Silva, Flavio M. Cárcano, Maurício G. Zaia, Luiz F. Lopes, Cristovam Scapulatempo-Neto, Céline Pinheiro

**Affiliations:** ^1^Molecular Oncology Research Center, Barretos Cancer Hospital, São Paulo, Brazil; ^2^Department of Pathology, Barretos Cancer Hospital, São Paulo, Brazil; ^3^Department of Medical Oncology, Barretos Cancer Hospital, São Paulo, Brazil; ^4^Barretos School of Health Sciences Dr. Paulo Prata—FACISB, São Paulo, Brazil; ^5^Barretos Children's Cancer Hospital, São Paulo, Brazil

**Keywords:** immunohistochemistry, metabolic reprogramming, testicular germ cell tumors, testicular neoplasms, Warburg effect

## Abstract

Testicular Germ Cell Tumors (TGCTs) are a rare group of neoplasms and the most common solid malignancy arising in young male adults. Despite the good response of these tumors to platinum-based chemotherapy, some patients are refractory to treatment and present poor clinical outcomes. During carcinogenesis and tumor development, cancer cells reprogram energy metabolism toward a hyper-glycolytic phenotype, an emerging hallmark of cancer. This phenomenon, known as the Warburg effect or aerobic glycolysis, involves overexpression of metabolism-related proteins, like glucose and monocarboxylate transporters, pH regulators and intracellular glycolytic enzymes. The metabolic profile of TGCTs is very little explored and, recently, this metabolic rewiring of cancer cells has been associated with aggressive clinicopathological characteristics of these tumors. The overexpression of monocarboxylate transporter 4 (MCT4) in TGCTs has been pointed out as a poor prognostic factor, as well as a promising therapeutic target. As a result, the main aim of the present study was to evaluate the prognostic value of key metabolism-related proteins in TGCTs. The immunohistochemical expressions of CD44 (as a monocarboxylate transporter chaperone), glucose transporter 1 (GLUT1), carbonic anhydrase IX (CAIX), hexokinase II (HKII) and lactate dehydrogenase V (LDHV) were evaluated in a series of 148 adult male patients with TGCTs and associated with clinicopathological parameters. In addition, paired normal tissues were also evaluated. The sample included 75 seminoma and 73 non-seminoma tumors. GLUT1 and CD44 expression was significantly increased in malignant samples when compared to paired normal samples. Conversely, HKII and LDHV expressions were significantly decreased in malignant samples. Concerning the clinicopathological values, CAIX expression was significantly associated with disease recurrence, while HKII expression was significantly associated with aggressive characteristics of TGCTs, including higher staging and non-seminoma histology. In conclusion, this study brings new insights on the metabolic characteristics of TGCTs, showing alterations in the expression of proteins related with the Warburg effect, as well as associations of the hyper-glycolytic and acid-resistant phenotype with aggressive clinicopathological parameters.

## Introduction

Testicular germ cell tumors (TGCTs) are the most frequent solid malignancies arising in young male adults (1, 2) and show an increase in incidence throughout the last decades, especially in Europea-descendent men (1, 3, 4). Divided into two major histological types, homogeneous seminoma and heterogeneous non-seminoma tumors (1), TGCTs tend to have a good response to platinum-based chemotherapy, with seminomas presenting more favorable outcomes in comparison to non-seminoma tumors (2). Despite the high rates of cure—over 90% in patients with early diagnosed disease (independent of histological type) (1, 5)—about 10–20% are refractory to treatment and present unfavorable clinical outcomes (6, 7).

The major mechanisms involved in the development of TGCTs are copy number variations (aneuploidies) and some recurrent somatic mutations. The isochromosome 12p is present in almost all tumors and is considered a marker for TGCTs. In fact, the underlying mechanisms involving isochromosome 12p in the development of TGCTs are still unclear, but there is enough evidence that implicate this alteration as an earlier triggering event, leading to invasiveness growth and malignization. Furthermore, the most frequently mutated driver oncogenes found in seminomas are *KIT* and *KRAS*, with 25–30 and 5–10% mutation frequencies, respectively (2, 4). Besides that, there is still a lack of information in understanding the complex heterogeneity of TGCTs, which highlights the importance of the discovery of different oncogenic events involving these tumors to optimize treatment and management. In this context, the recently described hallmark of cancer of deregulation of cellular energetics is gaining additional attention in the last years and should be considered as a possible relevant biological mechanism in TGCTs (8, 9).

During carcinogenesis and tumor development, cancer cells reprogram energy metabolism toward a hyper-glycolytic phenotype, even in the presence of high oxygen levels. This phenomenon, known as the Warburg effect or aerobic glycolysis, leads to a higher production of lactate than the normal metabolic phenotype, which relies mostly on oxidative phosphorylation (10–12). To fuel all the energy required and avoid intracellular acidification and apoptosis, cancer cells upregulate some key proteins, like glucose and monocarboxylate transporters, pH regulators and intracellular glycolytic enzymes (13). In comparison to oxidative phosphorylation, glycolysis is not an energetic efficient pathway but is a faster way to provide energy, metabolic intermediates, and biochemical building blocks, essential for anabolic reactions, and thus enhancing the aggressive characteristics presented by malignant cells (14). As demonstrated by many studies (15, 16), the overexpression of metabolism-related proteins plays an important role in the development and maintenance of the malignant phenotype of a vast majority of tumors. In this context, these metabolic players have been pointed out as prognostic factors and can be explored as promising therapeutic targets.

Although studies evaluating the implication of metabolic rewiring in the development and progression of TGCTs are lacking, there is evidence that these tumors present a highly glycolytic behavior, mainly attributed to their elevated levels of glucose consumption as demonstrated by ^18^F-fluorodeoxyglucose positron emission tomography (FDG-PET) studies (17–20). Furthermore, when comparing malignant and benign samples, a recent study (21) shows that TGCTs overexpress monocarboxylate transporter 4 (MCT4) and its chaperone CD147. Additionally, the upregulation of monocarboxylate transporter 1 (MCT1), MCT4 and CD147 has been associated with aggressive clinicopathological characteristics of these tumors, while MCT4 overexpression was associated with a worse prognosis, with patients presenting a shorter overall and event-free survival. Other studies show that TGCTs have an increase in the expression of glucose transporter 3 (GLUT3) protein (22), which is often found overexpressed by malignant neoplasms (23, 24).

Since metabolic reprogramming in human tumors need to be further exploited, the study of different metabolism-related proteins may offer a better understanding about their role, relevance, and implication in the biological characteristics and the complex heterogeneity of TGCTs. In this context, CD44, a chaperone for proper localization and activity of MCT1 and MCT4 in the plasma membrane, glucose transporter 1 (GLUT1), the pH regulator carbonic anhydrase 9 (CAIX), as well as hexokinase II (HKII), responsible for the irreversible glucose phosphorylation in the earlier steps of glycolysis and lactate dehydrogenase V (LDHV), the isoenzyme with higher affinity for pyruvate, that catalyze the conversion of pyruvate into lactate, arise as key players in the metabolic reprogramming of cancer cells (13, 16, 25, 26).

Therefore, regarding the emerging role of metabolic rewiring in tumors, as well as the biological complexity and the absence of studies considering this context in TGCTs, the aims of this study were to evaluate the expression of CD44, GLUT1, CAIX, HKII, and LDHV in TGCTs and normal samples, using tissue microarrays (TMAs), and to associate the expression with clinicopathological data to determine whether these proteins have some biological and/or prognostic value.

## Materials and Methods

### Case Selection and Clinicopathological Information

The series included 148 formalin-fixed paraffin embedded adult TGCTs samples, retrieved from the Pathology Department of Barretos Cancer Hospital, from 2007 to 2013. Only primary tumors, prior to chemotherapy, were selected. Additionally, paired normal samples were collected and analyzed when available (*n* = 66 for CD44, *n* = 59 for GLUT1, *n* = 78 for CAIX, *n* = 87 for HKII and *n* = 84 for LDHV; the different number of normal samples analyzed for each protein is related to sample loss as a result of block sectioning). The clinicopathological data included age, date of diagnosis, histological types, grading, staging (TNM), presence of vascular invasion, International Germ Cell Cancer Collaborative Group (IGCCCG) stratification risk (63), and dates of surgery, chemotherapy, recurrence, progression and death. Patients' mean age was 32.3 years (ranging from 18 to 73 years) and most of them were caucasian (62.2%). Detailed information on the clinicopathological data of the sample is depicted in Table 1. This study was approved by the Ethics Committee on Research of Barretos Cancer Hospital (number 541235).

**Table 1:** Clinicopathological characteristics of adult testicular germ cell tumor patients.

	***n* (%)**
**Histological type**	
Yolk sac	1 (0.7)
Choriocarcinoma	2 (1.4)
Embryonal carcinoma	8 (5.4)
Immature teratoma (grade I)	2 (1.4)
Mixed teratoma	19 (12.8)
Seminoma	75 (50.7)
Mixed germ cell tumor	41 (27.7)
**Stage at diagnosis**	
I	76 (51.3)
II	33 (22.3)
III	30 (20.3)
IS	7 (4.7)
**IGCCCG stratification risk**	
Low	43 (29.1)
Intermediate	17 (11.5)
High	7 (4.7)
**Chemotherapy**	
BEP	61 (41.2)
EP	14 (9.4)
Other	4 (2.7)
No chemotherapy	65 (43.9)
**Status—post treatment**	
Alive and disease free	128 (86.5)
Alive and in treatment	5 (3.4)
Cancer related death	12 (8.1)
Death from other causes	3 (2.0)

*BEP, bleomycin, etoposide, platinum; EP, etoposide, platinum; Other, carboplatin*.

### TMA Construction and Immunohistochemistry

TMAs were constructed for the immunohistochemical reactions. All the cases were reviewed by an experienced pathologist (ECAS) for diagnostic confirmation and demarcation of tumor areas for TMA cores. Each TMA contained sample cores of 1.0 mm diameter from all histological subtypes and corresponding normal tissues, in triplicate. Liver, kidney and placenta were used as controls for TMA orientation.

Immunohistochemistry for GLUT1 and CAIX was performed using a streptavidin-biotin-peroxidase complex (Ultravision Detection System: Large Volume Anti-Polyvalent, HRP, Lab Vision Corporation, Fremont, CA), according to manufacturer's instructions and as previously described (27). Immunohistochemistry for CD44 was performed using a biotin-free principle (ADVANCE HRP, Dako, Carpinteria, CA), according to manufacturer's instructions. For HKII and LDHV, the reactions were performed using an avidin-biotin-peroxidase complex principle (R.T.U. VECTASTAIN Kit, Vector Laboratories, Burlingame, CA), according to manufacturer's instructions. Details on antigen retrieval and each antibody used are described in Table 2. For visualization, slides were incubated with 3,3′-diamino-benzidine (Liquid DAB+ Substrate Chromogen System, Dako, Carpinteria, CA), according to manufacturer's instructions, then counterstained with hematoxylin and permanently mounted. As positive controls, placenta was used for GLUT1, normal gastric mucosa for CAIX and squamous cell carcinoma of oral cavity for CD44, HKII, and LDHV. Negative controls were available in the same tissue sections used as positive controls.

**Table 2:** Detailed aspects of immunohistochemistry.

**Protein**	**Antigen retrieval**	**Antibody**	**Clonality**	**Dilution, incubation time, and temperature**
CD44	Citrate (0.01 M, pH = 6.0), 98°C, 20 min	MCA2726 AbD Serotec	Monoclonal (156-3C11)	1:2000, 2 h, RT
GLUT1	Citrate (0.01 M, pH = 6.0), 98°C, 20 min	ab15309 Abcam	Polyclonal	1:500, 2 h, RT
CAIX	Citrate (0.01 M, pH = 6.0), 98°C, 20 min	ab15086 Abcam	Polyclonal	1:2000, 2 h, RT
HKII	EDTA (1 mM, pH = 8.0), 98°C, 20 min	ab104836 Abcam	Monoclonal (3D3)	1:1000, 2 h, RT
LDHV	EDTA (1 mM, pH = 8.0), 98°C, 20 min	ab101562 Abcam	Monoclonal (EPR1564)	1:6000, 2 h, RT

*RT, room temperature*.

### Immunohistochemical Evaluation

TMAs and whole sections were scored semi-quantitatively for extension of expression in cancer cells as follows: 0: no immunoreactive cells; 1: <5% of immunoreactive cells; 2: 5–50% of immunoreactive cells; and 3: >50% of immunoreactive cells. Also, intensity of staining was scored semi-qualitatively as follows: 0: negative; 1: weak; 2: intermediate; and 3: strong. The final score was defined as the sum of both parameters (extension and intensity) and grouped as negative (score 0–2) and positive (score 3–6), as previously described (28, 29). Only protein expression in plasma membrane was considered for CD44, GLUT1, and CAIX analysis, while for HKII and LDHV only cytoplasmic expression was considered for further analysis. TMAs were evaluated by two experienced pathologists independently (ECAS and CS-N). Discordant cases were reviewed and scored in consensus.

### Statistical Analysis

Data collected was analyzed using IBM SPSS Statistics software (version 23.0, IBM Company, Armonk, NY). During immunohistochemical evaluation, loss of tumor representativity in TMA cores as well as whole core loss was observed, influencing the final number of cases used for statistical analysis. Frequency of protein expression in normal and malignant tissues was compared using McNemar's test, while comparison with clinicopathological data was analyzed using Pearson's chi-square test and Fisher's exact test, according to the sample's characteristics. Overall survival was defined as the time from the date of primary diagnosis to last follow-up or death. Event-free survival was defined as the time from the primary diagnosis to the event date (recurrence, disease progression or death). None of the patients presented secondary tumors until the last follow-up. For survival models, only stage II and III patients were considered for further analysis. Overall and event-free survival curves were constructed using Kaplan-Meier's method and the data compared with log-rank test. Multivariate analysis by Cox proportional hazards regression model was used to determine independent predictors of survival. Independent variables were analyzed by univariate analysis, followed by multivariate analysis of all variables that reached a *p* < 0.2 at univariate analysis. For all tests, the level of significance established was 5% (significant results if *p* < 0.05).

## Results

### Expression of CD44, GLUT1, CAIX, HKII, and LDHV in Testicular Germ Cell Tumors and Paired Normal Tissues

**Figure 1:**
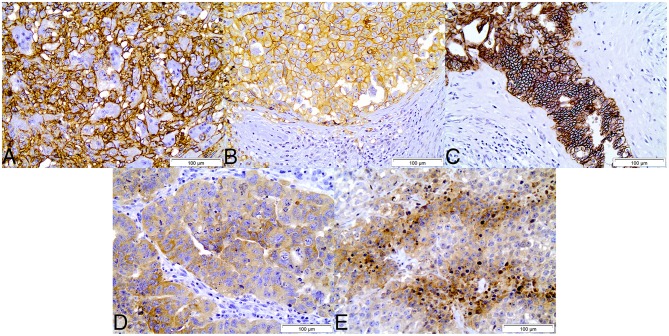
Immunohistochemical expression of CD44 **(A)**, GLUT1 **(B)**, CAIX **(C)**, HKII **(D)**, and LDHV **(E)** in adult testicular germ cell tumors. CD44, GLUT1, and CAIX show plasma membrane staining, while HKII and LDHV show cytoplasmic staining. Magnification: 400x.

Immuhistochemical evaluation of adult testicular germ cell tumors showed that expression of CD44, GLUT1 and CAIX was mostly exclusively found in plasma membrane. Regarding the expression of HKII and LDHV in tumor samples, both proteins were mostly detected in cytoplasm. According to the results observed in tumors, paired normal tissues showed similar expression patterns, with CD44, GLUT1 and CAIX frequently found in plasma membrane and HKII and LDHV in cytoplasm (Figure 1).

**Figure 2:**
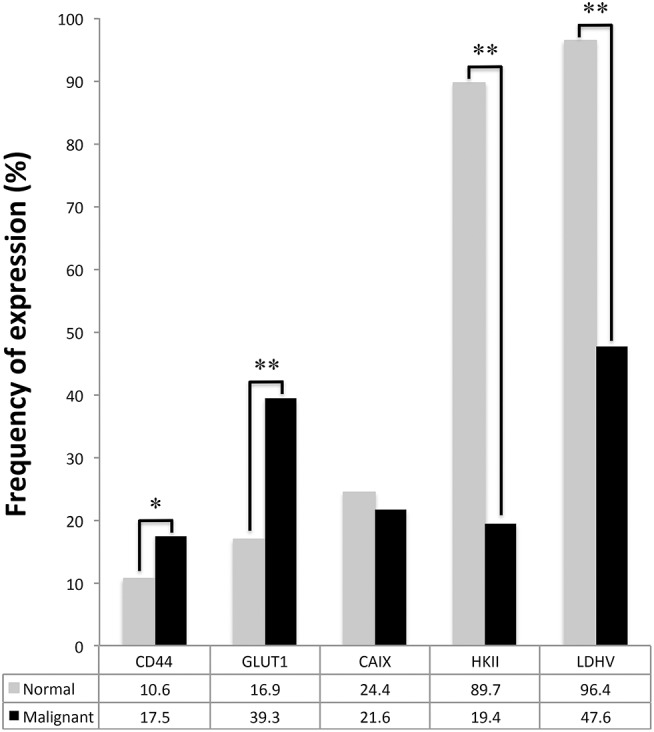
Plasma membrane expression of GLUT1, CAIX, and CD44 and cytoplasmic expression of HKII and LDHV in adult testicular germ cell tumors and correspondent normal testicular tissues. McNemar's test was used to assess differences of expression frequency between tumor and normal tissue. ^*^p = 0.004, ^**^p < 0.001.

Comparison of protein expression between tumor samples and paired normal tissues showed a significantly increased expression of CD44 and GLUT1 in tumor samples (*p* = 0.004 and *p* < 0.001, Figure 2). Conversely, HKII and LDHV expression was significantly decreased in malignant samples (*p* < 0.001 and *p* < 0.001, Figure 2).

### Clinicopathological Significance of Metabolism-Related Proteins

The associations between the expression of the metabolism-related proteins and the clinicopathological data of the patients are shown in Table 3. CAIX expression presented a significant association with recurrence (*p* = 0.024), while HKII showed a statistically significant association with non-seminoma tumors (*p* < 0.001) and higher stages (*p* = 0.019). CD44, GLUT1 and LDHV showed no significant associations with clinicopathological parameters.

**Table 3:** Association of CD44, GLUT1 and CAIX membrane expression and cytoplasmic expression of HKII and LDHV with clinicopathological characteristics of adult testicular germ cell tumors.

	**CD44**			**GLUT1**			**CAIX**			**HKII**			**LDHV**		
	***n***	**Positive (%)**	***p***	***n***	**Positive (%)**	***p***	***n***	**Positive (%)**	***p***	**n**	**Positive (%)**	***p***	***n***	**Positive (%)**	***p***
**Histology**			0.850			0.265^*^			0.367			**<0.001**			1.000
Seminona	54	24 (44.4)		68	63 (92.6)		59	23 (39.0)		73	13 (17.8)		73	64 (87.7)	
Non-seminomatous	60	25 (41.7)		72	70 (97.2)		66	32 (48.5)		71	34 (47.9)		72	63 (87.5)	
**T stage**			1.000			0.241^*^			0.465			0.363			0.064
T1	65	28 (43.1)		81	75 (92.6)		71	34 (47.9)		82	29 (35.4)		82	76 (92.7)	
T2+T3+T4	46	20 (43.5)		55	54 (98.2)		52	21 (40.4)		58	16 (27.6)		59	48 (81.4)	
**N stage**			0.701			0.238^*^			0.585			0.100			0.075
N0	61	27 (44.3)		79	73 (92.4)		71	29 (40.8)		81	21 (25.9)		81	67 (82.7)	
N1+N2+N3	51	20 (39.2)		58	57 (98.3)		52	24 (46.2)		60	24 (40.0)		61	57 (93.4)	
**M stage**			0.802			0.605^*^			0.209			0.457			0.738^*^
M0	94	40 (42.6)		114	107 (93.9)		104	42 (40.4)		120	37 (30.8)		119	103 (86.6)	
M1	18	7 (38.9)		24	24 (100.0)		19	11 (57.9)		22	9 (40.9)		24	22 (91.7)	
**Stage**			0.704			0.117^*^			0.715			**0.019**			0.078
I	54	24 (44.4)		71	65 (91.5)		63	26 (41.3)		73	17 (23.3)		73	60 (82.2)	
IS+II+III	57	23 (40.4)		66	65 (98.5)		59	27 (45.8)		68	29 (42.6)		69	64 (92.8)	
**Vascular invasion**			1.000			1.000^*^			0.693			0.428			0.236^*^
No	74	32 (43.2)		94	89 (94.7)		84	38 (45.2)		95	33 (34.7)		95	87 (91.6)	
Yes	34	15 (44.1)		39	37 (94.9)		37	15 (40.5)		42	11 (26.2)		43	36 (83.7)	
**IGCCCG stratification risk**			0.800^*^			1.000^*^			0.642^*^			0.470^*^			1.000^*^
Low	37	17 (45.9)		39	38 (97.4)		36	15 (41.7)		43	19 (44.2)		42	38 (90.5)	
Intermediate	15	5 (33.3)		17	17 (100.0)		15	8 (53.3)		17	6 (35.3)		17	16 (94.1)	
High	3	1 (33.3)		7	7 (100.0)		5	3 (60.0)		6	4 (66.7)		7	7 (100.0)	
**Recurrence**			0.551			0.598^*^			**0.024**			0.236^*^			0.219^*^
No	98	43 (43.9)		117	110 (94.0)		106	41 (38.7)		123	36 (29.3)		122	105 (86.1)	
Yes	12	4 (33.3)		16	16 (100.0)		14	10 (71.4)		15	7 (46.7)		16	16 (100.0)	
**Progression**			0.633^*^			1.000^*^			0.678^*^			0.662^*^			1.000^*^
No	58	25 (43.1)		64	63 (98.4)		59	28 (47.5)		68	24 (35.3)		68	60 (88.2)	
Yes	4	1 (25.0)		7	7 (100.0)		6	2 (33.3)		6	3 (50.0)		7	7 (100.0)	

* *Pearson's Qui-square test; Fisher's exact test. Significant results (p <0.05) are depicted in bold*.

### Survival Analysis

Kaplan-Meier analysis for the expression of metabolism-related proteins showed no significant associations with overall and event-free survival (data not shown). The predictive prognostic values of the proteins and clinicopathological parameters for overall survival and event-free survival were analyzed by means of Cox proportional hazards regression models (Tables 4, 5, respectively). Univariate analysis revealed predictive prognostic values for stage III and IGCCCG intermediate and high risk stratifications in overall and event-free survival. Multivariate analysis showed that IGCCCG high risk classification was an independent predictor for overall and event-free survival (HR: 9.987 *p* = 0.034 and HR: 11.061 *p* = 0.014, respectively). None of the analyzed proteins presented a prognostic value in TGCTs patients.

**Table 4:** Prognostic factors for overall survival in adult testicular germ cell tumors.

	**Univariate analysis**				**Multivariate analysis**			
	***n***	**HR**	**95% CI**	***p***	***n***	**HR**	**95% CI**	***p***
**Histology**								
Seminoma	26	1	-	-				
Nonseminomatous	37	0.980	0.340–2.826	0.971				
**Stage**								
II	33	1	-	-	33	1	-	-
III	30	5.166	1.436–18.587	0.012^*^	28	1.404	0.198–9.967	0.735
**Vascular invasion**								
No	35	1	-	-				
Yes	24	1.628	0.525–5.050	0.399				
**IGCCCG stratification risk**								
Low	37	1	-	-	37	1	-	-
Intermediate	17	4.344	1.036–18.215	0.045^*^	17	3.442	0.485–24.410	0.216
High	7	13.189	3.101–56.088	<0.001^*^	7	9.987	1.190–83.822	**0.034**
**CD44 plasma membrane**								
No	31	1	-	-				
Yes	20	1.040	0.293–3.693	0.951				
**CAIX plasma membrane**								
No	28	1	-	-				
Yes	24	0.963	0.294–3.161	0.951				
**HKII cytoplasm**								
No	36	1	-	-				
Yes	25	1.896	0.637–5.646	0.250				

*HR, hazard ratio; CI, confidence interval*.

* *Variables that reached p < 0.2 in univariate analysis. Significant results (p < 0.05) are depicted in bold*.

**Table 5:** Prognostic factors for event-free survival in adult testicular germ cell tumors.

	**Univariate analysis**				**Multivariate analysis**			
	***n***	**HR**	**95% CI**	***p***	***n***	**HR**	**95% CI**	***p***
**Histology**								
Seminoma	26	1	-	-				
Nonseminomatous	37	1.274	0.502-3.238	0.610				
**Stage**								
II	33	1	-		33	1	-	-
III	30	5.515	1.823–16.681	0.003^*^	27	1.517	0.277–8.317	0.631
**Vascular invasion**								
No	35	1	-	-				
Yes	24	1.121	0.426–2.953	0.817				
**IGCCCG stratification risk**								
Low	37	1	-	-	37	1	-	-
Intermediate	17	4.380	1.280–14.983	0.019^*^	17	3.222	0.203–6.954	0.176
High	7	16.230	4.483–58.758	<0.001^*^	6	11.061	1.627–75.201	**0.014**
**CD44 plasma membrane**								
No	31	1	-	-				
Yes	20	1.238	0.429–3.577	0.693				
**CAIX plasma membrane**								
No	28	1	-	-				
Yes	24	1.457	0.542–3.917	0.456				
**HKII cytoplasm**								
No	36	1	-	-	35	1	-	-
Yes	25	1.929	0.744–5.003	0.177^*^	25	2.015	0.738-5.500	0.172

*HR, hazard ratio; CI, confidence interval*.

* *Variables that reached p < 0.2 in univariate analysis. Significant results (p < 0.05) are depicted in bold*.

## Discussion

Our study showed an increase in the expression frequency of GLUT1 and CD44 in adult testicular germ cell tumors. In a previous study, the authors showed that MCT4 and CD147 expression was increased in TGCTs patients (21). Taken together, these results suggest a metabolic reprogramming of malignant cells toward a hyperglycolytic and acid-resistant phenotype in TGCTs.

The high frequency of GLUT1 expression is in agreement with the natural behavior of cancer cells as high glucose consumers. In fact, glucose transporters need to be overexpressed to fuel part of the metabolic reprogramming required by malignant cells to produce energy and intermediates for anabolism, as well as regulate redox state (30). This metabolic reprogramming demands an increased uptake of glucose, mainly provided by GLUT1 (31). In addition, CD44 expression is related to the overexpression of MCT4 previously described in TGCTs (21), suggesting that CD44 and MCT4 work together in lactate efflux (25), favoring the Warburg effect.

Regarding the association of metabolism-related proteins expression with clinicopathological characteristics of TGCTs, our study showed a significant association between CAIX status and recurrence, as well as HKII positive expression with aggressive clinicopathological parameters (non-seminoma tumors and higher staging). CAIX, a hypoxia marker, exerts a pH control regulation, which contributes to the acid-mediated cancer cell invasiveness when overexpressed (32–35), and has been associated with a worse prognosis in a variety of tumors (34). Also, the relation of CAIX expression with recurrence in TGCTs corroborates the role of CAIX in stimulating the invasion and aggressive phenotype of malignant cells and this association had been demonstrated by two studies (36, 37). In the firsty study (36), the authors show that CAIX serum levels was higher in TGCTs metastatic patients when compared to healthy controls, and that CAIX serum levels are significantly associated with intratumoral CAIX expression. However, CAIX serum levels do not demonstrate association with clinicopathlogical data, neither a prognostic value in clinical outcomes. According to the second study (37), the authors show a significant increase in the expression of CAIX in TGCTs samples compared to paired adjacent normal samples. This result, not reached by our study, probably can be explained by the numerical sample difference between the two works. Moreover, the authors show that CAIX positive expression was significantly associated with a worse progression-free survival, predominatly founded in patients with metastatic disease, which is in agreement with the association of CAIX positive expression with recurrence, demonstrated in the present study. Furthermore, the significant association of HKII positive expression with non-seminoma tumors and higher stages demonstrated by our study is in accordance with the role of this protein in providing energy for tumors, leading to disease progression and poor outcomes. Regardless of the lack of information about HKII expression in TGCTs patients, several studies associated HKII overexpression with aggressive characteristics and worse outcomes in different types of neoplasms, such as breast (38–40), cervical (41), colorectal (42), glioblastoma (43), liver (44, 45), lung (39), pancreatic (46, 47) and prostate (48). Indeed, HKII is described as one of the main proteins responsible for mediating the Warburg effect in cancer cells. This protein binds to the voltage dependent anion channel (VDAC) in mitochondria outer membrane, keeping the channel in an open state, gaining direct access to ATP generated intra-mitochondrially for glucose phosphorylation (49). This is in accordance with *in vitro* studies demonstrating the role of VDAC-bound HKII in supporting the Warburg effect (50). Additionally, other activities have been attributed to HKII in cancer metabolism context, which favors the aggressive phenotype of malignant cells, including the production of antioxidant molecules, direct protection of mitochondria against redox stress (anti-apoptotic effect) and facilitation of autophagy under starvation. Moreover, HIF-1α stimulation by AKT and mTORC1 has been described as the mechanism mainly responsible for HKII upregulation (51).

In contrast to GLUT1 and CD44 expression, a decreased frequency of HKII and LDHV expression in tumor samples, compared to normal testicular samples, was observed in the present study. Due to insufficient information available in the literature about the expression of HKII in normal testis, a search in the Human Protein Atlas (HPA) database, which integrates the protein expression profiles of 44 normal human tissues to RNA sequencing data of 32 out of these 44 tissue types (52), was conducted. HPA data showed that HKII RNA and protein expressions were correlated and more pronounced in male normal tissues, like testis and epididymis, corroborating our results. Also, a study done by Postic and collaborators (53), showed a relation between hexokinases and glucose transporters isoforms. Using rat models, the authors demonstrated that, during embryonic development, HKI and GLUT1 were the major isoforms expressed and related to energy production. After weaning, with the acquisition of insulin-sensitivity tissues, there is a switch in both isoforms, with HKII and GLUT4 participating mostly in energy production. These results suggest the important role of HKII in normal tissues after embryonic development for energy production. Our results are in accordance with this previous data as malignant testicular tissues tend to resemble the hypoxic and undifferentiated embryonic tissues (54, 55), therefore showing higher expression of GLUT1 but lower expression of HKII when compared to normal tissues. Regarding LDHV expression, Dodo and collaborators (56) found that LDHV was co-expressed with LDHX, the major LDH isoform present in testis, which has been found in different animals, including humans (57, 58). Curiously, LDHX mice knock-out presented an ablation of LDHV expression, not demonstrated by wild type mice, resulting in reduced energy production through glycolysis and impaired fertilization (56). Also, another study described that LDHV human transgene expression was able to restore LDHX expression in testis and sperm of LDHX knock-out mice, also restoring sperm motility and fertilization capacity (59). Taken together, these results suggest an important role of LDHV in aerobic glycolysis presented by normal testis, indicating that this protein is required to establish proper physiologic conditions for fertilization.

The natural behavior of cancer cells in reprogramming their metabolism, with a heavier reliance in aerobic glycolysis, provides a solid field for the development of anticancer therapies. Across the decades, different glycolytic inhibitors have been tested in pre-clinical studies and clinical trials, trying to kill cancer cells by pharmacological inhibition of glucose consumption and achieve therapeutic selectivity. Pelicano and co-workers (60) discuss about the use of three potent hexokinase inhibitors: 3-bromopiruvate (3-BP), 2-deoxyglucose (2-DG), and lonidamine. The major inhibitory mechanism of these compounds was the blockage of glucose phosphorylation, mediating the uncoupling of HK from mitochondria, leading to a rapid depletion of cellular ATP. Additionally, *in vitro* studies show a relevant therapeutic effect of the anti-hyperglycemic drug metformin in glycolytic addicted tumors, through the inhibition of HK function (38, 61). Finally, a recent study showed the efficacy of the antifungal drugs—ketoconazole and posaconazole—in glioblastoma cells, with selective inhibition of HKII. Using *in vitro* and *in vivo* models, the authors showed that both drugs were able to increase survival of mice and decrease cell proliferation and tumor metabolism (62). Importantly, both drugs are enrolled in an early phase I clinical trial in high grade gliomas (clinicaltrials.gov—NCT03763396). Despite the promising efficacy of the use of glycolytic inhibitors for glucose addicted tumors, TGCTs show a more complex biology. Our results demonstrate that HKII and LDHV have a role in normal testis, suggesting the importance of these markers in fertilization. Indeed, the use of HK inhibitors in TGCTs patients may lead to an increase in cytotoxicity and even infertility.

The present study, together with the study done by Silva and co-workers (21), corroborates that TGCTs present a switch in cellular metabolism toward a hyper glycolytic and acid-resistant phenotype, mainly associated with a worse prognosis. Additionally, HKII appears as a marker of tumor aggressiveness, bringing new insights about the metabolic characteristics of these tumors. Although our results showed a bone fide characterization of TGCTs metabolism, further studies are warranted to achieve a better understand, especially about HKII role in testicular malignancies, providing new evidences for future therapeutic strategies.

## Data Availability

The datasets generated for this study are available on request to the corresponding author.

## Ethics Statement

This study was approved by the Ethics Committee on Research of Barretos Cancer Hospital (number 541235). Written informed consent was waived as this study was considered a minimal-risk study.

## Author Contributions

MB performed immunohistochemical reactions, statistical analysis, and wrote the manuscript. MZ performed immunohistochemical reactions. ES and CS-N analyzed histological sections and performed the immunohistochemical evaluations. FC and LL aided in the study design and discussion of the results. CP was responsible for the study design, contributed to the discussion of the results, organization, and review of the manuscript. All authors read and approved the manuscript.

### Conflict of Interest Statement

The authors declare that the research was conducted in the absence of any commercial or financial relationships that could be construed as a potential conflict of interest.
